# Protocol for the measurement of changes in knowledge and engagement in the stepped wedge cluster randomised trial for childhood obesity prevention in Australia: (Reflexive Evidence and Systems interventions to Prevent Obesity and Non-communicable Disease (RESPOND))

**DOI:** 10.1186/s13063-020-04692-6

**Published:** 2020-09-04

**Authors:** Jillian Whelan, Claudia Strugnell, Steven Allender, Ariella R. Korn, Andrew D. Brown, Liliana Orellana, Josh Hayward, Vicki Brown, Colin Bell, Marj Moodie, Anna Peeters, Melanie Nichols

**Affiliations:** 1 Institute for Health Transformation, Global Obesity Centre, Deakin University, Geelong, Australia; 2 Friedman School of Nutrition Science and Policy, Tufts University, Boston, USA; 3 Biostatistics Unit, Deakin University, Geelong, Australia; 4 Institute for Health Transformation, Deakin Health Economics, Deakin University, Geelong, Australia; 5 Institute for Health Transformation, School of Health and Social Development, Deakin University, Geelong, Australia

## Abstract

**Background:**

Community-based interventions have shown promise in addressing the childhood obesity epidemic. Such efforts rely on the knowledge of key community members and their engagement with the drivers of obesity in their community. This paper presents the protocol for the measurement and evaluation of knowledge and engagement among community leaders within a whole-of-community systems intervention across 10 large intervention communities in Australia.

**Methods:**

We will investigate the role of stakeholder knowledge and engagement in the implementation and effectiveness of the stepped wedge cluster randomised trial in ten communities in Victoria, Australia. Data will be collected using the Stakeholder-driven Community Diffusion Survey (SDCD) to measure levels of knowledge and engagement prior to commencement (2019), across the three separate levels of governance within the intervention at five time points. Primary outcomes will be baseline overall knowledge and engagement scores across the three levels of governance and change in overall knowledge and engagement over time.

**Discussion:**

We hypothesise there will be heterogeneity between intervention sites on levels of knowledge and engagement and that these differences will be associated with variability in implementation success.

**Trial registration:**

Australian New Zealand Clinical Trials Registry ACTRN12618001986268. Registered on 11 December 2018

## Introduction

### Background

Addressing childhood obesity is an international priority [1]. Within Australia, almost 25% of Australian children aged 5–17 experienced overweight or obesity in 2017–2018 [2]. There is evidence that the actual prevalence may be far higher than national surveys with relatively low response rates [3]. It is clear that childhood obesity significantly impacts quality of life and mental health [4]. As childhood obesity tracks into adulthood [5], leading to ever more serious health consequences, there is an urgent need to intervene early if the health and social and economic impacts of obesity are to be addressed [6].

A major challenge to the prevention of childhood obesity is the complexity arising from its multiple interdependent and systemic causes, ranging from individual biology through to environmental drivers of food and physical activity choices [1]. The importance of a systems approach to addressing childhood obesity is supported by the existing trial evidence that suggests multi-component and multi-level interventions that increase community capacity and include both healthy eating and physical activity are more likely to have a positive impact [7]. Several systematic reviews have highlighted that the prevention of childhood obesity is possible through comprehensive community-based interventions [8–10] and engaged leadership and relevant support structures and capacity [11]. Noteworthy interventions have included Shape Up Somerville in the USA [12]; Be Active Eat Well [13], It’s Your Move [14] and Romp & Chomp [15] in Australia; Fleurbaix and Laventie in Northern France [16]; and Children’s Healthy Living [17] in the Pacific region.

Consistent with the need to address complexity, Swinburn et al. [18] pointed to a third generation of community-based intervention trials that explore what works within different system contexts and how existing systems can be strengthened to prevent disease. There are now several major examples internationally of large scale interventions meeting this challenge, intervening across multiple communities and taking an explicitly complex systems approach to engaging communities and catalysing action [19–22]. Such approaches have potential to optimise implementation approaches because they emphasise “…capacity building, creativity and innovation, relationships, engagement, communication, embedded action and policies, robustness and sustainability, facilitative leadership, and embedded monitoring and evaluation” [23] p. 2.

Intervening at a different level necessitates different tools to evaluate the effectiveness of these actions. The tools required to measure these elements have evolved over time. Community capacity measures focus primarily on the four domains of leadership, resources, partnership and intelligence [24], and community readiness, measures that explore the five domains of knowledge of the issue, awareness of efforts to address the issue, community climate, leadership and resources [25]. The common elements within these tools emphasise the importance of knowledge, resources and leadership. These constructs, although shown to relate to successful obesity prevention [26], are difficult constructs to measure [27] and were not specifically designed to measure changes in underlying determinants of obesity over time.

Korn et al. [28] developed a tool that includes the major concepts we aim to measure in systems change: knowledge, resources and leadership, and extended the relevance to systems change by looking at the specific characteristics that leaders require to catalyse change and diffuse innovation effectively throughout the community with a specific focus on the prevention of childhood obesity. The resulting Stakeholder-driven Community Diffusion (SDCD) Survey assesses several domains which quantifies the knowledge about the problem of obesity, knowledge about effective and sustainable interventions, available resources, mutual learnings, flexibility, leadership and trust (defined as belief and confidence in others). This tool aligns with previous attempts to measure concepts of readiness and capacity but refines these through an emphasis on understanding the drivers of success in community-based system-level interventions and a focus on core elements identified in the literature that diffuse innovation and catalyse change through communities.

#### Problem statement

Despite increasingly complex intervention approaches, there has been little progress in understanding how community leaders’ knowledge, engagement and social networks contribute to the success or otherwise of community-based interventions. This is partly due to a historical focus on evaluating outcomes from single community trials, with less emphasis on the specific characteristics that may lead to successful outcomes within these trials and across broader multi-community trials.

This paper presents the protocol for the measurement and evaluation of knowledge and engagement among community leaders’ for the Reflexive Evidence and Systems interventions to Prevent Obesity and Non-communicable Disease (RESPOND) trial, a whole-of-community systems intervention across 10 large intervention communities (local government areas (LGAs)) in Victoria, Australia. This protocol follows the SPIRIT checklist for guidance in the reporting of protocols for clinical trials [29]. The measurement of leaders’ knowledge and engagement over multiple time points, along with intervention process measures, will enable investigation of the following research questions:

- RQ1: What is the baseline level of community leaders’ knowledge and engagement in each of the governance and implementation groups within the project and how does it differ between communities?
- RQ2: How do levels of knowledge and engagement among community leaders change during participation in a whole-of-community systems intervention?
- RQ3: To explore whether knowledge and engagement levels, and/or changes in knowledge and engagement levels, predict implementation effectiveness of system-level intervention?

Our hypothesis is that there will be heterogeneity between intervention sites on baseline levels of knowledge and engagement and that these differences will be associated with variability in implementation success and level of intervention within a system. The level of system intervention actions will be evaluated against the Meadows framework [30]. This assessment includes, for example, changes to a variable, connections between these variables, rules governing the system and goals of the system [30].

## Methods

RESPOND utilises systems approaches in the design, implementation and evaluation of a large-scale intervention to prevent obesity among children aged 0–12 years. It has prospective registration as a clinical trial [31], and is a stepped-wedge cluster randomised trial in 10 communities. This protocol explains one sub-study of the RESPOND trial that relates to changes in knowledge and engagement.

**Fig. 1:**
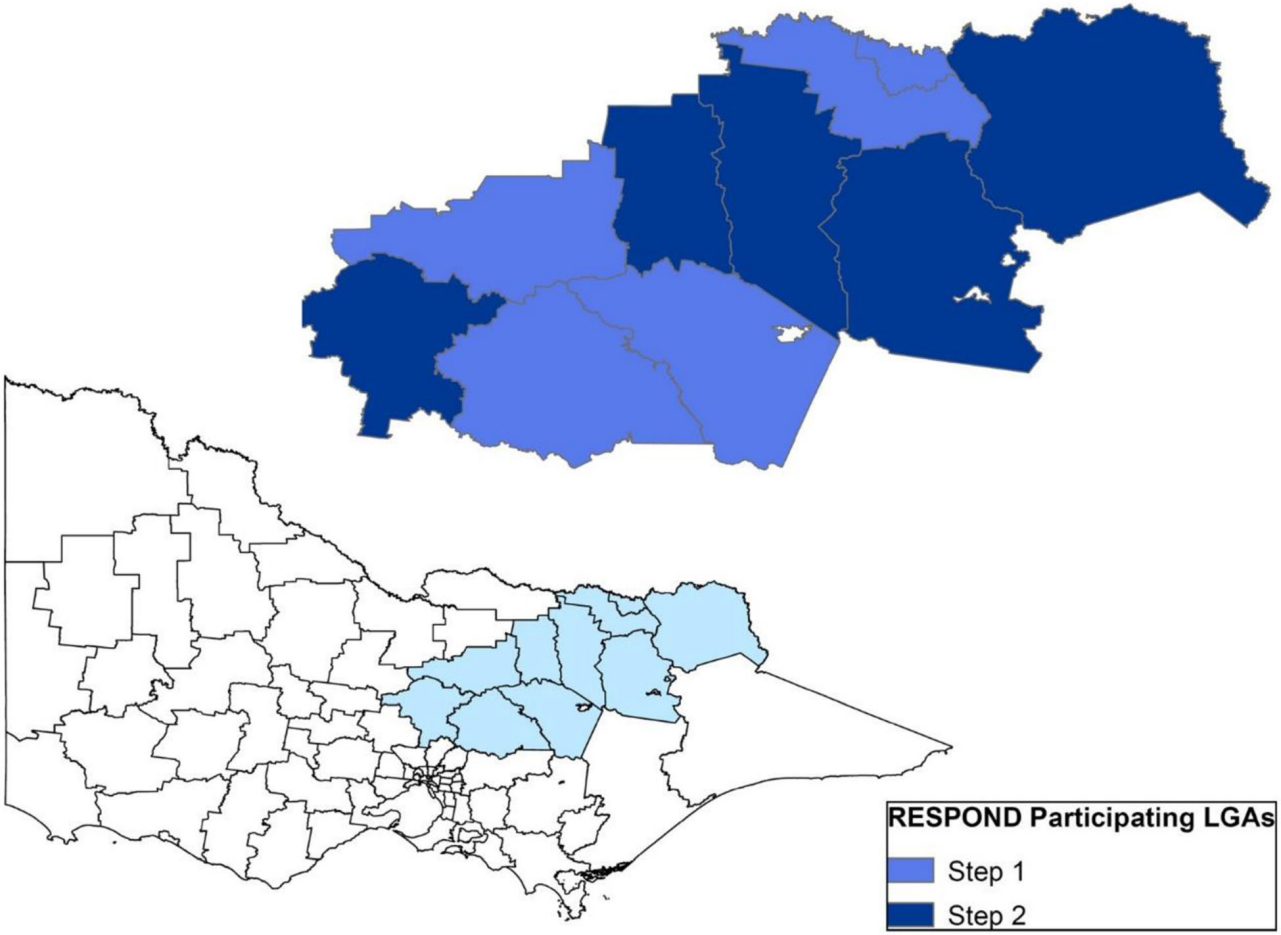
Trial setting: Ovens Murray and Goulburn regions of north eastern Victoria, Australia

The setting for the trial is the Ovens Murray and Goulburn regions of north eastern Victoria, Australia Fig. 1, which covers a population of approximately 30,000 children (aged 0–12 years) [32]. For the purpose of trial design, communities are defined as local government areas (LGAs), which are the geographical divisions administered by municipal governments within Australian states and the Northern Territory [33]. The unit of randomisation and intervention (clusters) is the LGA (rather than individual children or townships). The ten LGAs will be ranked in order of population size and divided in five pairs. A computer-generated random list will be generated by the study statistician who will not be involved with any aspect of the enrolment process and has no contact with the communities. One community from each pair will be randomly allocated to receive the intervention at step 1. Blinding is not feasible in this trial as the whole community is recruited into the design, implementation and evaluation.

The primary outcome for RESPOND is a change in children’s standardised body mass index (BMIz) among primary school-aged children in Grade 2 (aged approx. 7–8 years), Grade 4 (aged approx. 9–10 years) and Grade 6 (aged approx. 11–12 years). Secondary outcome measures include modifiable obesogenic risk factors of children (physical inactivity, sedentary behaviour, poor diet quality, poor sleep and health-related quality of life) and wellbeing of children attending schools in participating communities, at each of three measurement waves (June 2019, 2021 and 2023).

### Participants

**Fig. 2:**
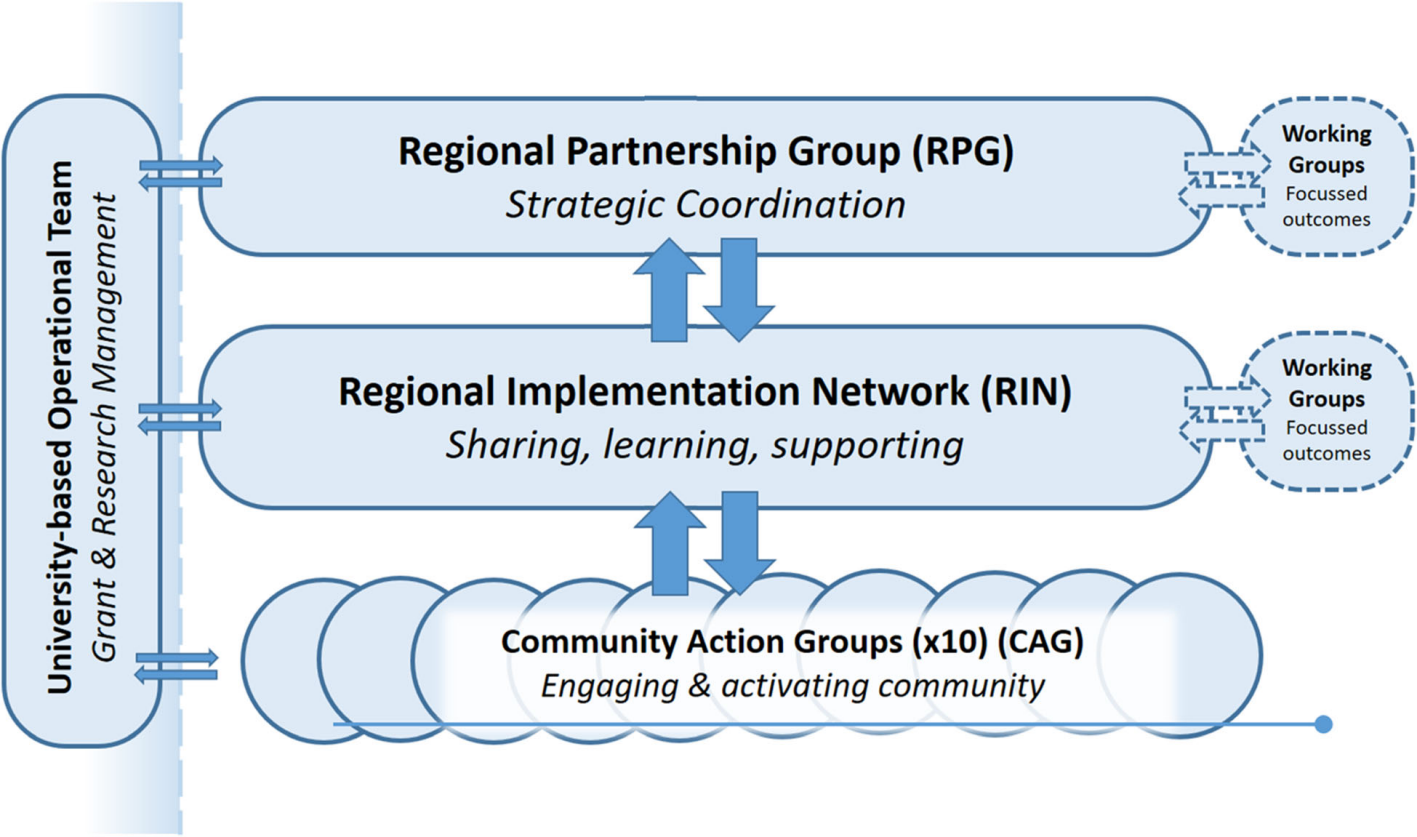
The RESPOND Governance Structure

Members of each of the groups within the RESPOND governance structure (as shown in Fig. 2) will be invited to participate in the Knowledge & Engagement Survey. This includes a single overarching Regional Partners Group (RPG) which comprises senior executive representation from all partners to the National Health and Medical Research Council (NHMRC) grant. This to date includes in alphabetical order: Beechworth Health Service, Central Hume Primary Care Partnership, Gateway Health, Goulburn Valley Primary Care Partnership, Greater Shepparton City Council, Lower Hume Primary Care Partnership, Numurkah District Health, Upper Hume Primary Care Partnership, VicHealth, the Victorian Department of Education and Training, the Victorian Department of Health and Human Services and Yarrawonga Health. This group provides overarching direction and governance of the RESPOND project. The Regional Implementation Network (RIN) comprises operational and co-ordinator level staff from each of the partner agencies (listed above), along with representation from each of the implementation communities (who are not necessarily signed partners to the grant). This network provides connections and knowledge exchange between the RPG and the individual communities and provides a forum for learning and sharing of experience among the individual communities. The third group comprises the 10 community action groups (CAG), one within each community whose role is to coordinate the implementation of local intervention activities. Participants will be invited to participate in the trial at each data collection point. Any contact details obtained at baseline or at follow-up data collection points will be retained subject to our ethics approvals and used for follow-up data collection. We will send three reminders to request participants to participate in data collection.

The number of possible participants will be used as the denominator and is based on maximum possible participation across all tiers of the governance structure and implementation teams (Table 1).

**Table 1:** Sampling frame: groups and estimated maximum respondent numbers per community

Governance groups	Members	Number of groups	Estimated* maximum possible participants per group	Estimated* maximum possible number of responses per time point	Proposed timelines
Regional Partners Group (RPG)	Signatories to the funded grant	1	44	44	Feb/Mar 2019–2023
Regional Implementation Network (RIN)	One prevention staff member from each community (*n* = 10)	1	10	10	Feb/Mar 2019–2023
Community Action Groups (CAG)	Participants of three workshops and community-led strategies	10	100	1000	Feb/Mar 2019–2023

*Estimated maximum participant numbers based on the current number of 12 partner organisations which may increase or decrease throughout the life course of the grant

Including baseline, for the Knowledge and Engagement Survey, there are five data collection points over the 5-year period of the grant in step 1 communities, and three data collection points in step 2 communities (this survey is administered annually). It is theoretically possible that a maximum of 4270 observations could be collected.

### Intervention

The RESPOND intervention involves catalysing systems change through community-led collective action. It comprises four main components adapted to be delivered at-scale from the process described by Sweeney et al. [34]. RESPOND aims to engage whole communities in a locally led multifaceted response. Implementing the four components of the intervention is intended to disrupt the current trajectory of obesity prevalence by instigating actions around the modifying determinants of childhood obesity (e.g. physical activity, sedentary behaviour, poor diet quality and sleep insufficiency). In brief, the four components include the following (Table 2):

**Table 2:** Four components of the RESPOND intervention

Component	Description
Component 1: Catalyse and Set up	Establishment of strong governance within partnership group with clearly defined roles and responsibilities of the partners. Identification of capacity for component 2.
Component 2: Monitoring	Routine childhood obesity and risk factor surveillance/monitoring system. Data will be collected every 2 years.
Component 3: Community engagement	Local leaders receive relevant training in group model building and facilitation.
Component 4: Implementation and diffusion	Implementation of community-led actions and networking opportunities between communities.

The intervention comprises four components: Within the first component (Catalyse and set up), strong governance will be established within the partnership group, and the roles and responsibilities of different partners will be established. Routine meetings of the partnership governance group will be established to maintain engagement and prioritisation of the intervention. To catalyse and prepare for the commencement of the intervention at each step, key partners in each region will identify capacity to support the monitoring and community engagement components described below.

The second component (Monitoring) will be a routine childhood obesity and risk factor surveillance/monitoring system to be established across the 10 LGAs, with data collection repeated every 2 years. Data collection is supported by locally based prevention capacity identified in component one. In addition to supporting overall evaluation of RESPOND, key statistics on children’s weight status and health behaviours will be provided to support the community engagement component—providing impetus for community action and context around the current state of child health in the communities.

In the third component (Community engagement), local leaders receive training in group model building (GMB) and facilitation techniques. Recruitment to these training sessions were snowballed from local leaders (identified in the first component). Attendance is regularly encouraged to maximise adherence to the prescribed GMB process during delivery. Following training, facilitators will recruit local leaders and champions into group model building workshops to develop a causal loop diagram (CLD) of the communities perceived drivers of childhood obesity, as they apply to their community and context. Following the development of the CLD, facilitators will support community leaders and champions to design intervention actions to be implemented and led by the community.

Within component four (Implementation and diffusion), community-led actions are implemented. Community stakeholders will share with the researchers the ‘actions’ identified within their group model building workshops. Alongside implementation, active communities will be connected with each other and with the research team via monthly meetings as an ‘implementation network’. This network will form a forum for communities to share learnings on effective strategies to support and guide community actions as they emerge. The research group will assist the stakeholders to identify effective strategies to support local actions, but will not directly participate in the operational management of actions. Reminders will be sent at least 1 week prior to the event for all training and workshop activities and attendance registers will be kept.

The explanation above outlines the intervention, and more detail is available in the trial registration [31]. The exploration of the change in knowledge and engagement over time, as outlined in this protocol, is one key element of this study. The intervention has been funded through an Australian National Health and Medical Research Council (NHMRC) Partnership Project grant (1151572), in partnership with 12 organisations; partners have committed > $3.5 m—a mix of staff time and cash contributions. Ethics approval has been obtained from Deakin University Faculty of Health Human Ethics Advisory Group (HEAG-H 173_2018) and for the childhood obesity monitoring system by Deakin University’s Human Research Ethics Committee (2018-381), the Victorian Department of Education and Training (2019-003943) and the Catholic Archdiocese of Melbourne and Sandhurst. All invited participants will provide informed consent prior to gaining access to the online survey. There will be no special criteria for discontinuing or modifying allocated interventions.

### Outcomes

The SDCD will measure levels of knowledge and engagement prior to commencement, during intervention (annually, 2019–2023) and at the end of the intervention (2023). The Regional Partners Group and Implementation Network will be surveyed at all five time points, while the community-specific Community Action Groups will be surveyed annually from the time the groups come into existence post GMB 3 (2019 for step 1, 2021 for step 2).

#### Primary outcomes

1. Baseline overall knowledge and engagement scores across the three levels of governance: Regional Partners Group, Regional Implementation Network and Community Action Groups.
2. Change in overall knowledge and engagement since baseline across the three levels of governance.

#### Secondary outcomes

1. Changes in knowledge and engagement sub-scales over time since baseline across the three levels of governance between 2019 and 2023.
2. Association between baseline and/or changes in knowledge and engagement with the type and level of community-led actions matched against the variables and connections identified in the causal loop diagrams created. For example, active travel as a variable may result in specific actions such as the construction of bike storage or bus drop-off zones to improve active travel.

### Recruitment

The survey will be completed online, and all potential participants will receive an email link to the survey. The Regional Partner Group will be directly invited to participate by the researchers, through contact lists for the RESPOND project quarterly meetings, which are convened and chaired by the University-based operational team (Fig. 2). The project partner organisations are responsible for convening the RIN and CAG; therefore, through third party recruitment, these partner organisations will send the survey invitations to all participants at those levels so that the researchers and partner agencies do not break the Victorian Privacy and Data Protection Act 2014 [35]. Based on previous experience of our group in similar surveys, we anticipate a response rate of 65% for the RPG and RIN and a response rate of 40% for the CAG.

### Data collection

Data will be collected using a modified version of the Stakeholder-driven Community Diffusion Survey (SDCD) developed by Korn et al. [28]. This survey developed by Korn et al.’s [28] was informed by extensive work with communities implementing interventions using a stakeholder-driven community diffusion model [12] and an in-depth review of the core elements of capacity building that are specific to community-based participatory childhood obesity prevention interventions [11]. This work identified strong community engagement as being associated with positive intervention outcomes, and the development of effective coalitions in turn built community capacity, particularly in the fields of leadership [28]. This tool is designed to be simple to administer and capture the elements of community capacity that are directly relevant to the community and systems-based approach and childhood obesity focus of the RESPOND intervention.

Our modified survey consists of 43 questions across five knowledge domains and five engagement domains (see Additional file 1). The networks components of the SDCD survey will be omitted. The data will be collected via the online survey platform Qualtrics [36] and takes 10–15 min to complete. Respondents are asked to answer knowledge and engagement questions on a five-point scale: strongly disagree, disagree, neutral, agree, strongly agree. High levels of knowledge are characterised by an understanding of obesity prevention at the community scale, encompassing knowledge of the problem, intervention factors, stakeholder roles, sustainability and resources. A person with little understanding of these factors would likely have no or low levels of relevant knowledge, which would be expected to impede diffusion of an evidence-informed intervention through a community. High levels of engagement represent positive characteristics on the five domains of dialogue and mutual learning (for example, openness and collaborative behaviours), flexibility, influence and power, leadership and trust (for example, high levels of trust in colleagues and commitment to promoting a climate of trust and collaboration). Across the 10 domains, the survey aims to efficiently capture all relevant domains of community capacity that are hypothesised to influence intervention outcomes and/or be impacted by intervention implementation. Limited personal data will be collected, such as age and years of experience in the field, and a unique identifier will be assigned during analysis to enable tracking of responses over time.

The survey was minimally modified for the RESPOND intervention to ensure appropriateness to the local context and intervention. Our intervention specifically relates to children aged under 12 years, so the phrase ‘age range 0 to 12 years’ was added. Two further questions related to resources were added, based on a recent systematic review of sustainability of community-based obesity prevention interventions where ‘resources’, both human and financial, were identified as the most cited reason for sustainability of interventions [37]. These two questions were pilot tested with the RESPOND Regional Partner Group in December 2018 and wording was edited until the group agreed that the meaning was clear. This amended SDCD is available in Additional file 1 (additional items are questions 16 and 17 in Domain 5: Available resources). More detail is shown in Table 3.

**Table 3:** Knowledge and engagement survey structure

Survey structure	No. of questions
**Section 1 Knowledge**	
Domain 1: The problem of childhood obesity	5
Domain 2: Modifiable determinants of childhood obesity (intervention factors)	3
Domain 3: Sustainability	3
Domain 4: Stakeholders’ roles	3
Domain 5: Resources	5
**Section 2: Engagement**	
Domain 1: Dialogue and mutual learning	7
Domain 2: Flexibility	3
Domain 3: Influence and power	2
Domain 4: Leadership and stewardship	10
Domain 5: Trust	2
**TOTAL**	**43**

### Data management and analysis plan

The responses to each SDCD survey question will be coded from 0 (strongly disagree) to 1 (strongly agree) [0 = 0 strongly disagree, 0.25 = disagree, 0.50 = agree, 0.75 = agree, 1.00 = strongly agree]. Domain summaries are the average of items within each domain. Participant summaries are averages of the domains (domain-weighted rather than item-weighted). We view the domains as equally important as each other, and the number of items is not related to how important the domains are, rather the complexity of eliciting the information about each domain. The SDCD survey will be summarised at participant level as the average of items within each of the five domains of knowledge (5 knowledge sub-scores) and the five domains of engagement (5 engagement sub-scores) (see Table 3).

- *Analysis*: RQ1: What is the baseline level of community leaders’ knowledge and engagement in each of the governance and implementation groups within the project and how does it differ between communities?

Variability of scores and sub-scores of knowledge and engagement at baseline across participant communities will be estimated based on surveys completed by participants in the Local Implementation Groups. Of note, ‘baseline’ corresponds to the 2019 survey for step 1 communities and to the 2021 survey for step 2 communities. Variability across communities will be estimated using a linear mixed model (multi-level) with community as the random factor.

- *Analysis*: RQ2: How do levels of knowledge and engagement among community leaders change during participation in a whole-of-community systems intervention?

We will fit linear mixed models with time as fixed effect and participant as random effect (to account for the repeated measurements) to assess whether a temporal trend exists in the SDCD scores (and sub-scores) and whether the intervention induces changes in trends of SDCD scores. For Regional Implementation Network participants/communities, the model will further include community allocation to step 1 or 2 as fixed effect, and the breakpoint (2021) to allow for changes in slope in step 2 communities. For Local Implementation Groups, the model will additionally include community as a random effect to account for clustering. If for any participant group a linear relation between a given score and time is not supported by the data, time will be incorporated as a categorical variable.

- *Analysis*: RQ3: Explore whether average community baseline knowledge and engagement levels and/or average community changes in knowledge and engagement levels are associated with implementation effectiveness of system-level intervention.

For each community, data on knowledge and engagement of community leaders at baseline and changes in knowledge and engagement will be summarised using location and dispersion measures. We will explore whether these measures are associated with implementation effectiveness (number of actions implemented by the community) using nonparametric correlation and if appropriate linear models.

## Discussion

The SDCD survey utilised in this study will provide unique targeted insights across multiple dimensions of knowledge and engagement in this scaled up, systems level, whole of community obesity prevention intervention. Similar studies have measured related concepts of community readiness through more labour intensive tools such as the community readiness to change tool [25] and have linked these changes to reductions in obesity prevalence [26].

The system-level nature of RESPOND provides opportunities not only for the application of existing learnings to apply a multi-strategic approach to community prevention [10] but also provides opportunity to test new and emerging system-level evaluation strategies such as social network analysis [38] to both supplement and better understand changes in health behaviours and obesity prevalence within such scaled-up interventions [19].

### Data monitoring

The data monitoring committee comprises the grant Chief Investigator team (annual meetings) and the RESPOND Project Management team (fortnightly meetings). Interim results will be disseminated in draft form to our intervention communities to assist with planning of relevant intervention activities. All results will be aggregated to LGA level or higher, and data from any communities with low response rates will be additionally checked before preparation of results materials to ensure that no individual or organisation is potentially identifiable. Only the research staff approved on the ethics application will have access to the raw data. There are no expected serious adverse effects that are detrimental to the community stakeholders. However, the RESPOND Operational team will monitor the progress of the trial, and if any adverse events eventuate, these will be discussed with Deakin University’s Human Research Ethics committee appropriate actions including cessation. Annual reports will routinely be submitted to relevant ethics committees.

### Dissemination policy

Results from this study will be published in peer-reviewed manuscripts and will be presented to local community groups and stakeholders, national and international conferences as relevant. The authorship guidelines [39] will be followed for all relevant publications and presentations. Open access publication of this protocol will facilitate full public access to our protocol.

## Conclusion

We hypothesise that community-led systems childhood obesity prevention efforts require significant knowledge and engagement from community stakeholders to be effective and sustainable. This study will add to the evidence base that seeks to better understand the role of community characteristics in the successful implementation of complex interventions. We will examine changes over time in these key variables and the associations, if any, with the level of activity and implementation of prevention strategies within communities.

## Trial status

Protocol Version 1, October 2019.

Recruitment for step 1 communities commenced in April 2019 and is expected to be completed by December 2019. Recruitment for step 2 communities will commence in February 2021 and is expected to be completed by June 2021.

**Fig. 3:**
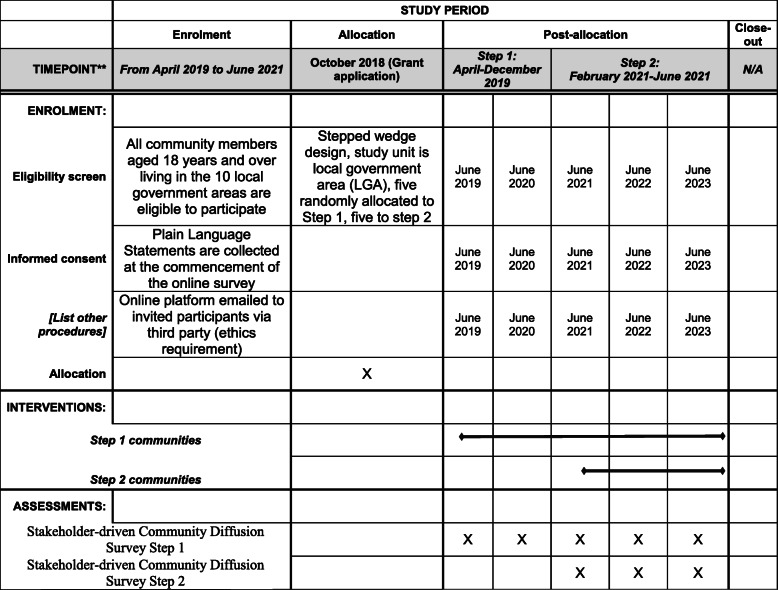
Schedule of enrolment, interventions and assessments

Figure 3 shows the SPIRIT schedule of enrolment, interventions and assessment relevant to this study protocol.

## Supplementary information

**Additional file 1.** RESPOND Knowledge and Engagement Survey.

## Data Availability

Supporting data for the protocol include the survey which is attached as supplementary material. The de-identified datasets collected and analysed during this proposed study will be available on request from the corresponding author subject to the publications arising from the dataset having being published.

## Abbreviations

RESPOND: Reflexive Evidence and Systems interventions to Prevent Obesity and Non-communicable Disease
SDCD: Stakeholder-driven Community Diffusion Survey
LGAs: Local government areas
BMIz: Children’s standardised body mass index
RPG: Regional Partners Group
NHMRC: National Health and Medical Research Council
RIN: Regional Implementation Network
CAG: Community action groups
GMB: Group model building
CLD: Causal loop diagram
