# Supplementary material for: Protocol for the measurement of changes in knowledge and engagement in the stepped wedge cluster randomised trial for childhood obesity prevention in Australia: (Reflexive Evidence and Systems interventions to Prevent Obesity and Non-communicable Disease (RESPOND))
Source: Trials. 2020 Sep 4;21:763. doi: 10.1186/s13063-020-04692-6 (PMC7650215; doi:10.1186/s13063-020-04692-6)
Supplement: Supplementary file 1 — Additional file 1. RESPOND Knowledge and Engagement Survey. [file 13063_2020_4692_MOESM1_ESM.docx]

**Appendix I. RESPOND Knowledge and Engagement Survey**

Thank you for your participation in the RESPOND Knowledge and Engagement study. This survey will take approximately 6 minutes of your time. If you’d like to take a break from the survey, your responses will automatically be saved when you exit the page. To return to the survey, re-click the link found in your email and you will be brought to the question where you last left off. Please note that there is a bar at the top of the screen that shows your progress with the survey from 0 to 100%. All responses will be kept confidential, and only research staff will have access to the responses.

Are you aged 18 years or older?

Yes
No

(If no, the online survey skips automatically to the end of the survey.)

This survey includes questions on the following topics:

1. Your perspectives and understanding of early childhood* obesity prevention
2. Your engagement with the issue, which includes your involvement, adaptability, influence, leadership, and trustworthiness
3. Demographic information

*When we refer to **childhood**, we mean children ages 0 to 12 years.

Please type in your name in the space below and then click “begin” to start the survey:

**Section 1 [KNOWLEDGE]**

The following questions will ask you about your perspectives and understanding of early childhood obesity prevention in your local government area.

To what extent do you agree or disagree with the following statements?

|  | | Strongly disagree  (1) | Disagree  (2) | Neutral  (3) | Agree  (4) | Strongly agree  (5) |
| --- | --- | --- | --- | --- | --- | --- |
| **Domain 1: The problem of childhood obesity (Problem)** | | | | | | |
| 1 | Early childhood obesity is a problem in my local government area |  |  |  |  |  |
| 2 | There are increased health care costs due to medical complications of obesity in childhood |  |  |  |  |  |
| 3 | I am familiar with other illnesses and health concerns associated with obesity in childhood |  |  |  |  |  |
| **Domain 2: Modifiable determinants of childhood obesity and level of social ecology to address them (Intervention factors)** | | | | | | |
| 4 | Certain populations of children are more vulnerable to obesity |  |  |  |  |  |
| 5 | I feel confident in listing risk factors related to childhood obesity |  |  |  |  |  |
| 6 | I am aware of evidence-based strategies that target risk factors related to childhood obesity |  |  |  |  |  |
| **Domain 3: Stakeholders’ roles in the whole intervention, what others are doing, and knowledge of multi-setting components (Roles)** | | | | | | |
| 7 | I can play a role in preventing childhood obesity in my local government area |  |  |  |  |  |
| 8 | I know what is being done in the my local government area community to prevent childhood obesity |  |  |  |  |  |
| 9 | Obesity prevention strategies should be prioritized and implemented by multiple childhood providers |  |  |  |  |  |
| **Domain 4: How to intervene to achieve sustainability (Sustainability)** | | | | | | |
|  | I know strategies to prevent obesity in childhood that… |  |  |  |  |  |
| 10 | Will be acceptable and appropriate for the my local government area community |  |  |  |  |  |
| 11 | Will have the greatest impact in promoting healthy weight |  |  |  |  |  |
| 12 | Can be sustained over time |  |  |  |  |  |
| **Domain 5: Available resources (Resources)** | | | | | | |
| 13 | I know where to find resources related to childhood obesity prevention |  |  |  |  |  |
| 14 | I am aware of staff education or training opportunities that include the subject of childhood obesity prevention |  |  |  |  |  |
| 15 | I am aware of evaluation and monitoring efforts in my local government area that address childhood obesity in young children |  |  |  |  |  |
| 16 | Within our organisation we have paid staff who can be involved in the RESPOND project. |  |  |  |  |  |
| 17 | Our community has volunteers who can be involved in the RESPOND project |  |  |  |  |  |
| **Domain 1 (continued): What level to intervene upon and which modifiable determinants of childhood obesity to address (Intervention factors)** | | | | | | |
| 17 | I feel confident in listing possible policy changes to combat childhood obesity in my local government area |  |  |  |  |  |
| 18 | I feel confident in suggesting changes in childhood settings (like child care, medical settings, or play areas) to prevent childhood obesity |  |  |  |  |  |

**Section 2 [ENGAGEMENT]**

This section of the survey will ask you about your *engagement* with the topic of childhood obesity prevention in my local government area. In this study, we refer to *engagement* as an individual’s sense of involvement, adaptability, influence, leadership, and trustworthiness. In addition, we use the term *colleagues* in this survey to mean coworkers (e.g., staff and supervisors/managers) and peers in the community (e.g., collaborators and fellow committee or coalition members).

To what extent do you agree or disagree with the following statements?

|  |  | Strongly disagree  (1) | Disagree  (2) | Neutral  (3) | Agree  (4) | Strongly agree  (5) |
| --- | --- | --- | --- | --- | --- | --- |
| **Domain 1: Dialogue & mutual learning** | | | | | | |
| 1 | I can talk openly and honestly at work or meetings |  |  |  |  |  |
| 2 | I make an effort to participate in discussions |  |  |  |  |  |
| 3 | I listen to colleagues when someone expresses a concern |  |  |  |  |  |
| 4 | I am attentive to what colleagues say when they speak |  |  |  |  |  |
| 5 | I share my ideas and suggestions whether or not colleagues agree with my input |  |  |  |  |  |
| 6 | I can openly discuss problems and issues |  |  |  |  |  |
| 7 | I work with colleagues to develop the best possible approach to our work |  |  |  |  |  |
| **Domain 2: Flexibility** | | | | | | |
| 8 | I am willing to make compromises related to my work in childhood obesity prevention |  |  |  |  |  |
| 9 | I work to come up with solutions that satisfy all colleagues |  |  |  |  |  |
| 10 | I respect different points of view from colleagues |  |  |  |  |  |
| **Domain 3: Influence & power** | | | | | | |
| 11 | I influence decisions that affect childhood obesity prevention efforts in the community |  |  |  |  |  |
| 12 | I influence the policies and actions of community-based childhood obesity prevention |  |  |  |  |  |
| **Domain 4: Leadership & stewardship** | | | | | | |
| 13 | I am motivated to prevent childhood obesity |  |  |  |  |  |
| 14 | I establish positive relationships with community members with whom my colleagues want to engage and mobilize |  |  |  |  |  |
| 15 | I have good skills for working with other people and organizations |  |  |  |  |  |
| 16 | I lead by example |  |  |  |  |  |
| 17 | I encourage colleagues to express their opinions and thoughts |  |  |  |  |  |
| 18 | I emphasize the importance of having a collective sense of mission |  |  |  |  |  |
| 19 | I provide leadership and guidance in maintaining relationships among colleagues |  |  |  |  |  |
| 20 | I advocate strongly for my own opinions and agendas |  |  |  |  |  |
| 21 | I do not give up when faced with challenges |  |  |  |  |  |
| 22 | I try to promote a climate of collaboration and trust |  |  |  |  |  |
| **Domain 5: Trust** | | | | | | |
| 23 | I trust others involved in childhood obesity prevention efforts |  |  |  |  |  |
| 24 | People involved in childhood obesity prevention efforts trust me |  |  |  |  |  |

In this section, you will be asked questions about yourself, including questions about your employment, age and experience.

What is your age?

|  | 18-24 (1) | 25-29 (2) | 30-34 (3) | 35-39 (4) | 40-44 (10) | 45-49 (5) | 50-54 (6) | 55-59 (7) | 60-64 (8) | 65 and over (9) |
| --- | --- | --- | --- | --- | --- | --- | --- | --- | --- | --- |
| Please select your age grouping |  |  |  |  |  |  |  |  |  |  |

How many years of experience do you have in this field?

|  | 0 | 1 | 2 | 3 | 4 | 5 | 6 | 7 | 8 | 9 | 10 | 11 | 12 | 13 | 14 | 15 | 16 | 17 | 18 | 19 | 20 |
| --- | --- | --- | --- | --- | --- | --- | --- | --- | --- | --- | --- | --- | --- | --- | --- | --- | --- | --- | --- | --- | --- |

| Please use the slider to indicate your years of experience in this field () | 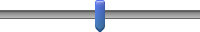 |
| --- | --- |

Please select the group you are **most closely** affiliated with when working with children aged 0-12

- Primary Care Partnership (1)
- Early education (2)
- Child care (3)
- Health care (4)
- Schools (5)
- Health department (6)
- Education Department (7)
- Community-based organisation, please provide details (8) ______________________________
- Your local government area, please provide details (9) _________________________________
- Parent (10)
- Community member (11)
- University (12)
- Other, please provide details (13) ________________________________________________

If applicable, please tell us your title for the group/organization/department chosen above:
 ________________________________________________________________

Besides [above affiliation] do you have a secondary affiliation with another group that works with children aged 0-12 years?

- Yes (1)
- No (2)
- Primary Care Partnership (3)
- Early education (4)
- Child care (5)
- Health care (6)
- Schools (7)
- Health department (8)
- Education Department (9)
- Community-based organisation, please provide details (10) ________________________
- Your local government area, please provide details (11) ____________________________
- Parent (12)
- Community member (13)
- University (14)
- Other, please provide details (15) ________________________________________________

Please select the secondary group you are affiliated with when working with children aged 0-12

- Primary Care Partnership (1)
- Early education (2)
- Child care (3)
- Health care (4)
- Schools (5)
- Health department (6)
- Education Department (7)
- Community-based organisation, please provide details (8) ______________________________
- Your local government area, please provide details (9) _________________________________
- Parent (10)
- Community member (11)
- University (12)
- Other, please provide details (13) ________________________________________________

If applicable, please tell us your title for the group/organisation/department chosen above

________________________________________________________________

You will be invited to complete this survey six-monthly over the next five years. If you agree to participate in future surveys, please provide your name and an email address so we can send the invitation to participate and the survey to you in approximately six months time.

________________________________________________________________

If you have any comments or queries to add to this survey about any of the topics covered, please enter this here.

________________________________________________________________

Thank you, you have now answered all our questions. Thank you for participating in the survey! If you have any questions or concerns about this survey or the study in general, please email [details removed]. When you proceed to the next screen your answers will be submitted.

Regards
